# Studying intramuscular fat deposition and muscle regeneration: insights from a comparative analysis of mouse strains, injury models, and sex differences

**DOI:** 10.1186/s13395-024-00344-4

**Published:** 2024-05-29

**Authors:** Alessandra M. Norris, Kiara E. Fierman, Jillian Campbell, Rhea Pitale, Muhammad Shahraj, Daniel Kopinke

**Affiliations:** Department of Pharmacology and Therapeutics, Myology Institute, University of Florida, Gainesville, FL USA

## Abstract

Intramuscular fat (IMAT) infiltration, pathological adipose tissue that accumulates between muscle fibers, is a shared hallmark in a diverse set of diseases including muscular dystrophies and diabetes, spinal cord and rotator cuff injuries, as well as sarcopenia. While the mouse has been an invaluable preclinical model to study skeletal muscle diseases, they are also resistant to IMAT formation. To better understand this pathological feature, an adequate pre-clinical model that recapitulates human disease is necessary. To address this gap, we conducted a comprehensive in-depth comparison between three widely used mouse strains: C57BL/6J, 129S1/SvlmJ and CD1. We evaluated the impact of strain, sex and injury type on IMAT formation, myofiber regeneration and fibrosis. We confirm and extend previous findings that a Glycerol (GLY) injury causes significantly more IMAT and fibrosis compared to Cardiotoxin (CTX). Additionally, females form more IMAT than males after a GLY injury, independent of strain. Of all strains, C57BL/6J mice, both females and males, are the most resistant to IMAT formation. In regard to injury-induced fibrosis, we found that the 129S strain formed the least amount of scar tissue. Surprisingly, C57BL/6J of both sexes demonstrated complete myofiber regeneration, while both CD1 and 129S1/SvlmJ strains still displayed smaller myofibers 21 days post injury. In addition, our data indicate that myofiber regeneration is negatively correlated with IMAT and fibrosis. Combined, our results demonstrate that careful consideration and exploration are needed to determine which injury type, mouse model/strain and sex to utilize as preclinical model especially for modeling IMAT formation.

**Supplementary Information:**

The online version contains supplementary material available at 10.1186/s13395-024-00344-4.

## Introduction

Skeletal muscle is the largest organ in the body, comprising between 30–40% of our body mass, and is essential for life and longevity [1–4]. Muscle mass and function can be severely compromised in certain diseases such as muscular dystrophies [5–7], neuromuscular diseases [8, 9], and diabetes [10–12], as well as in sarcopenia [13, 14]. A shared hallmark of these conditions is the progressive loss of muscle tissue and its replacement with intramuscular fat (IMAT) and fibrotic scar tissue, together called fatty fibrosis [15–18]. While numerous efforts are focused on preserving muscle mass, no therapeutic interventions exist to limit the progressive infiltration of IMAT.

The mouse, *Mus musculus*, is the most prolific model organism for studying human physiology and disease [19], and is the predominant model organism for pre-clinical studies [20], due to its many advantages such as its high percentage of shared genome, easy handling and maintenance, ability to genetically modify and its comparable short life span. For example, the *mdx* mouse carries a point mutation in the dystrophin genes, the same gene that is disrupted in Duchenne muscular dystrophy (DMD) patients [21–24]. Therefore, *mdx* mice have been widely used as a popular preclinical model to study DMD. However, while DMD patients have a progressive loss of muscle mass and subsequent replacement with IMAT [25], *mdx* mice on a C57BL/10 background lack key disease characteristics such as loss of muscle and increased fibrosis and IMAT [26, 27]. The DBA/2 strain has been shown to be a superior model than the C57BL/10 strain by having increased fibrosis, degeneration of muscle and reduced lifespan [26–28]. This highlights the strong influence strains have on phenotypes. In addition, despite its prominence in disease, IMAT has remained a mostly understudied feature due to the lack of an appropriate mouse model. To determine the most suitable mouse strain to study IMAT, we sought to compare muscle regeneration, IMAT formation and fibrosis between female and male C57BL/6J (Bl6), 129S1/SvlmJ (129S) and CD1 mice. Not only are these commonly used strains, but both Bl6 and 129S are isogenic strains, meaning they are considered genetically identical within each other; while the CD1 strain is an outbred strain, where high genetic diversity is obtained. Isogenic and outbred strains have their advantages and disadvantages that should be taken into consideration to what best fits the experimental design and area of research. In our laboratory, we have utilized all three previously mentioned mouse strains and have qualitatively noticed vast differences in IMAT infiltration, leading us to quantitively compare IMAT, muscle regeneration and fibrosis across different strains, injury models and sex.

We induced muscle injuries in both females and males, through intramuscular injections of Cardiotoxin (CTX) and Glycerol (GLY), two commonly used injury models. CTX is a myotoxin derived from cobras, such as *Naja pallida*, that is believed to specifically cause myofiber damage and produce little IMAT, leaving other compartments unharmed [29, 30]. While little is understood about GLY, we and others have shown that this injury model causes significant IMAT formation and is, therefore, considered an adipogenic injury model [17, 31–39]. Our main aim in this study is to answer the impact 1) mouse strains, 2) sex and/or 3) different injury models have on IMAT, myofiber regeneration and fibrosis.

We found that Bl6 mice are the most resistant to IMAT compared to 129S and CD1 mice, which display ample IMAT formation. We also found sex differences in IMAT formation where females of all strains had a higher adipogenic capacity than males. GLY also induced more fibrosis compared to a CTX injury, except for 129S mice, which, interestingly, are the most resistant to injury-induced fibrosis. When assessing muscle regeneration, Bl6 completely recovered myofiber size 21 days post injury. In contrast, 129S and CD1 mice did not fully recover their myofibers to pre-injury levels, highlighting the high regenerative capacity of Bl6 mice. When evaluating the potential relationship between IMAT and muscle regeneration, we found an inverse correlation between the amount of IMAT and myofiber size post injury. The same was true for fibrosis. Lastly, we tested the impact of genetic background on IMAT by intercrossing both isogenic mouse strains, Bl6 and 129S, and found an intermediary phenotype in the hybrid B6129SF1/J mouse. This suggests that relatively few crosses might be needed to significantly increase IMAT compared to the pure Bl6 strain. Together, our findings highlight the importance of choosing the correct mouse strain, sex and injury model to study human pathological features in the mouse.

## Results

### Comparing Bl6, 129S and CD1 strains in an uninjured setting

**Fig. 1:**
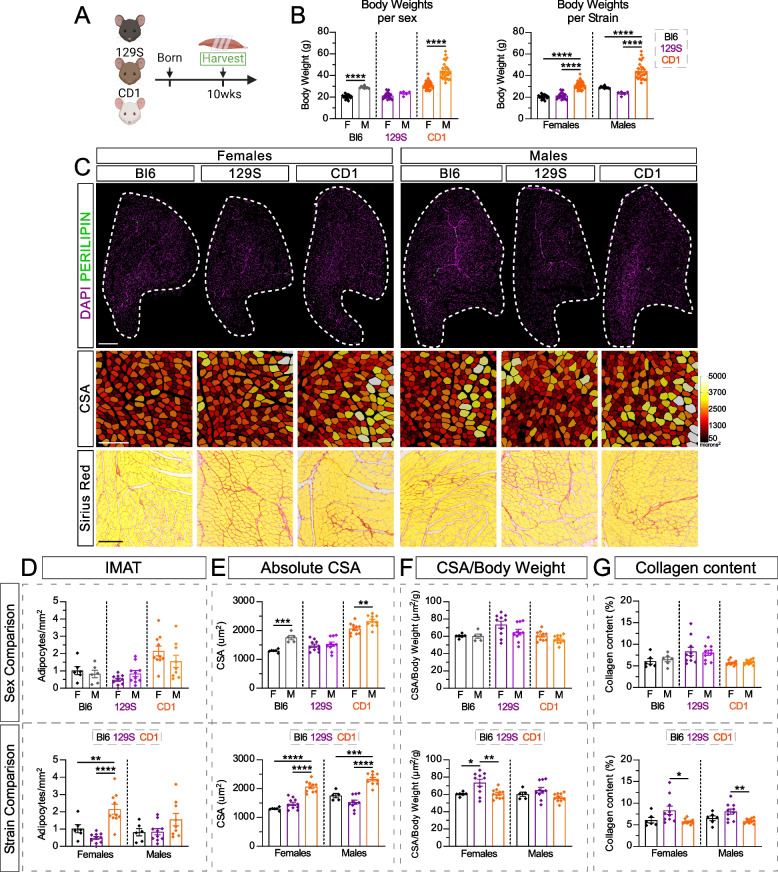
Comparison of IMAT, myofiber size and collagen deposition between mouse strains and sex during muscle homeostasis. **A** Experimental outline. **B** Body weights (g) of male (M) and female (F) mice from C57BL/6J (Bl6), 129S1/SvlmJ (129S) & CD1 strains at 10 weeks of age. Data are grouped to compare between sexes within the same strain *(Left)*; and within the same sex *(Right)*. **C** *Top*: Immunofluorescence of Tibialis Anterior muscles (TAs) of uninjured Bl6, 129S & CD1 females and males to visualize PERILIPIN^+^ adipocytes (green). Nuclei are marked by DAPI (magenta). Scale bar: 500 µm. *Middle*: Individual muscle fibers, stained by PHALLOIDIN and false color-coded according to size (µm^2^). Scale bar: 250 µm. *Bottom*: Collagen deposition (red) is detected by the histological stain Sirius Red. Scale bar: 250 µm. **D** Quantification of IMAT normalized to area of uninjured TA (adipocytes/mm^2^). **E** Average cross-sectional area (CSA) of total myofibers (µm^2^). **F** Average CSA of total myofibers normalized to body weight (µm^2^/g). **G** Quantification of area occupied by collagen deposition normalized to total TA area (%). **D-G** *Top*: Data are grouped to compare between sexes within the same strain. *Bottom*: Data are grouped to compare between strains within the same sex. All data are represented as mean ± SEM. An unpaired two-tailed t test or a one-way ANOVA followed by a Dunnet’s multiple comparison was used. A p value less than 0.05 was considered statistically significant where: * *p* ≤ 0.05, ** *p* ≤ 0.01, *** *p* ≤ 0.001 and **** *p* ≤ 0.0001

We first compared the general differences between two commonly used inbreed strains, C57BL/6J (Bl6) and 129S1/SvlmJ (129S), and the outbred strain CD1 by assessing body weight, IMAT, collagen content and cross-sectional area (CSA) of myofibers in males and females (Fig. 1A). At 10 weeks of age, males were significantly heavier than females in both the Bl6 and CD1 strains, while there were no sex differences in body weight in the 129S strain (Fig. 1B). When comparing amongst strains, CD1 mice were the heaviest in both sexes, while 129S and Bl6 displayed similar body weights (CD1 > Bl6/129S; Fig. 1B).

Intramuscular adipose tissue, also known as intramuscular fat (IMAT), was quantified by counting individual adipocytes marked by PERILIPIN followed by normalization to the total area of the cross-section of the Tibialis Anterior (TA) (Fig. 1C). As expected, a young and healthy murine muscle contains very little IMAT. Interestingly, while we found no sex differences within the three strains, CD1 females displayed two-fold more IMAT compared to Bl6 and 129S females and all males (Fig. 1D).

We next evaluated the differences in myofiber size between strains and sexes. Cross-sections of TAs were stained with PHALLOIDIN, marking F-actin, to visualize individual fibers and their size was measured through our previously published pipeline, utilizing the deep-learning segmentation algorithm Cellpose, followed by our plugin LabelsToRois [33]. Consistent with body weights, we found that males had a larger average CSA than females in both CD1 and Bl6 strains (M >> F), while there was no difference in average CSA between sex in the 129S strain (M = F; Fig. 1E). Additionally, CD1s had the highest CSA compared to the remaining strains in both sexes (CD1 >> Bl6/129S; Fig. 1E). The difference in average CSA is further appreciated when assessing the size distribution of all fibers (% of total fibers), where CD1 mice displayed a rightward shift of their fiber distribution (i.e. fewer small-sized and more large-sized fibers) in comparison to 129S and Bl6 mice (Supplemental Fig. 1A and B).

Taking into consideration the significant differences in body weight between strains and sex, and how increased body weight can impact myofiber size [40], we normalized the average CSA to the individual’s body weight (µm^2^/g). After normalizing CSA to body weight, all previous sex differences in CSA (Fig. 1E) disappeared (Fig. 1F). Thus, body weight has a clear impact on myofiber size. When comparing between strains, 129S females, but not males, had the highest CSA to body weight ratio (Fig. 1F).

Lastly, we compared the amount of extracellular matrix present prior to any injury by measuring the area occupied by collagen (% of total TA area) through the histological stain Sirius red (Fig. 1C). We only observed slightly higher collagen content in both female and male 129S mice compared to Bl6 and CD1 mice (129S > Bl6 = CD1; Fig. 1G).

Taken together, a young and healthy muscle only contains minute amounts of IMAT with only CD1 females displaying slightly higher levels. Interestingly, it appears that the heavier the mouse, the more hypertrophied their myofibers are, which can be accounted for when normalizing to body weight. For example, CD1 mice are the heaviest mouse strain with the largest average CSA. However, once the CSA is normalized to body weight, CD1 mice are comparable to the rest of the strains. Thus, normalizing CSA measurements can allow for a more direct comparison of mice that display differences in body weight. To note, this is not taking into consideration any potential strain- or sex-specific differences, as well as other features of muscle architecture such as fiber number and pennation angle.

### IMAT varies between strains and sex but is dependent on injury model

**Fig. 2:**
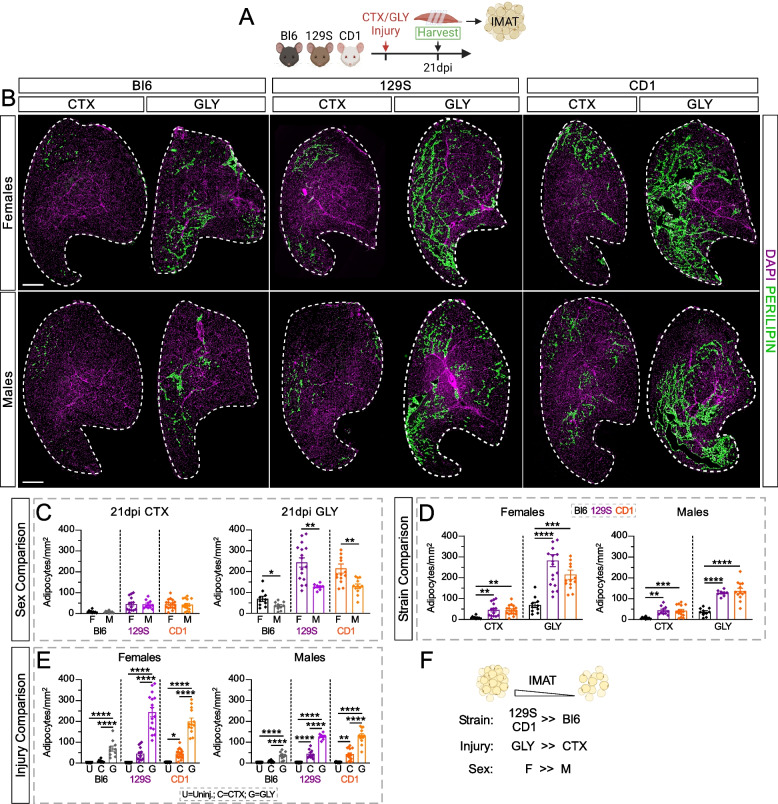
IMAT infiltration post injury differs between strains, sexes, and injury types. **A** Experimental outline. **B** Immunofluorescence of mature adipocytes (PERILIPIN^+^ cells; green) 21 days post injury (dpi) with Cardiotoxin (CTX) or Glycerol (GLY), in females (F) and males (M) from C57BL/6J (Bl6), 129S1/SvlmJ (129S) & CD1 mouse strains. Nuclei are visualized through DAPI (magenta). Scale bar: 500 µm. **C-E** Quantification of adipocytes normalized to injured area (adipocytes/mm.^2^) 21 days after CTX or GLY injury in male (M) and female (F) Bl6, 129S & CD1 mice. Data are grouped to compare between: **C** sexes within the same injury and strain; **D** strains within the same injury and sex; **E** injuries within the same sex and strain. **F** *Summary model*: 129S and CD1 strains have more IMAT than Bl6; GLY induces more IMAT than CTX; and females have more IMAT than males. All data are represented as mean ± SEM. An unpaired two-tailed t test or a one-way ANOVA followed by a Dunnet’s multiple comparison was used. A p value less than 0.05 was considered statistically significant where: * *p* ≤ 0.05, ** *p* ≤ 0.01, *** *p* ≤ 0.001 and **** *p* ≤ 0.0001

Even though mice are extremely lean compared to humans, IMAT can be induced in mice through injury models, such as Glycerol (GLY), and to a lesser extent, Cardiotoxin (CTX). To determine whether Bl6, 129S and CD1 strains have different susceptibility to IMAT formation, adult 10-week-old female and male Bl6, 129S and CD1 mice were injured with either CTX or GLY and IMAT was evaluated 21 days after injury (Fig. 2A). IMAT was quantified by counting individual PERILIPIN^+^ adipocytes (Fig. 2B) followed by normalization to the total injured area of the cross-section of the TA. Interestingly, while we failed to observe any sex differences post CTX injury, females of all three strains had significantly higher IMAT after a GLY injury (F >> M; Fig. 2B and C), suggesting sex differences that are dependent on injury type.

When assessing strain differences, we discovered that Bl6 mice formed the least amount of IMAT following both injuries. In contrast, CD1 and 129S mice, while comparable between each other, developed 3–sixfold more IMAT (Bl6 <<< 129S/CD1; Fig. 2D). Thus, Bl6 mice are highly resistant to IMAT formation. Finally, when comparing the effects of injury type on IMAT formation, we found that GLY induces significantly higher amounts of IMAT across all strains and both sexes compared to CTX, demonstrating that GLY is a strong adipogenic inducer (GLY >>> CTX; Fig. 2E).

As adipocytes can vary widely in size, we also assessed differences in the size of individual adipocytes (µm^2^) between sex, strains, and injury type. We did not observe any sex dependent differences in adipocyte size after a CTX injury (F = M; Supplemental Fig. 2A). However, 129S males had larger adipocytes than females after a GLY injury (M > F; Supplemental Fig. 2A). Therefore, adipocyte size does not largely vary between sexes after injury. When comparing between strains, CD1 females had the largest adipocyte size after both CTX and GLY injuries (CD1 > Bl6/129S; Supplemental Fig. 2B). Within males, adipocyte size did not vary after a CTX injury, but after a GLY injury 129S males had larger adipocytes than Bl6 males (GLY: 129S > Bl6; Supplemental Fig. 2B). We next compared adipocyte size between injury types. While only significant for some (female CD1 and male 129S), a GLY injury appears to cause adipocytes to be larger compared to CTX (Supplemental Fig. 2C).

Taken together, we find that Bl6 are the most resistant to injury-induced IMAT compared to 129S and CD1 strains, a GLY injury causes significantly more IMAT to a CTX model across all strains and females exhibit more IMAT than males after a GLY injury (Fig. 2F). In contrast to the total amount of adipocytes, adipocyte size appears to be only slightly affected by strain and/or sex. However, adipocytes tend to be larger post GLY than CTX injury.

### Fibrotic scaring varies within strains and injury models, while displaying no sex difference

**Fig. 3:**
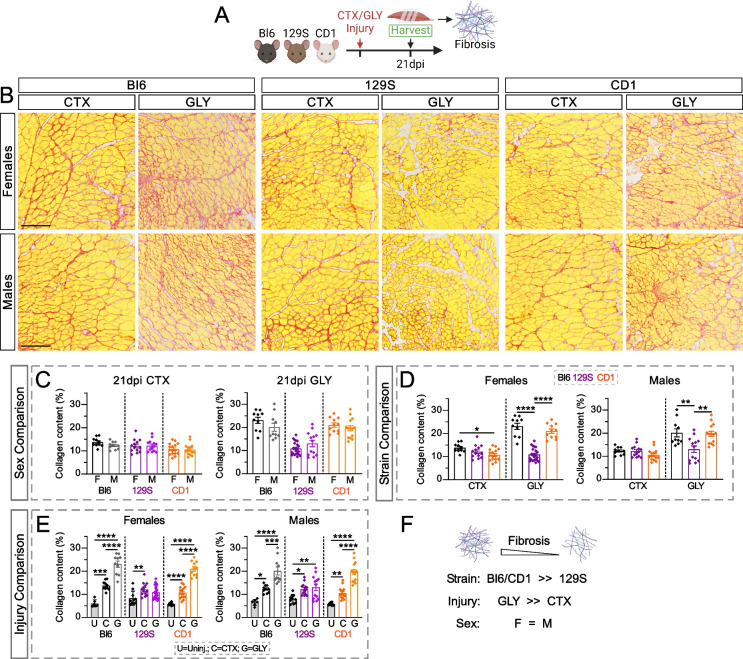
Tissue fibrosis is mostly affected by strain and injury type but independent of sex. **A** Experimental outline. **B** Collagen deposition (red) of Tibialis Anterior (TA) cross sections was visualized by the histological stain Sirius Red 21 days post Cardiotoxin (CTX) or Glycerol (GLY) injury in C57BL/6J (Bl6), 129S1/SvlmJ (129S) & CD1 female (F) and male (M) mice. Scale bar: 500 µm. **C-E** Quantification of area occupied by collagen deposition normalized to total TA area (%). Data are grouped to compare between: **C** sexes within the same injury and strain; **D** strains within the same injury and sex; **E** injuries within the same sex and strain. **F** *Summary model*: Bl6 and CD1 strains are more fibrotic than 129S; and GLY induces a higher fibrotic response than a CTX model. All data are represented as mean ± SEM. An unpaired two-tailed t test or a one-way ANOVA followed by a Dunnet’s multiple comparison was used. A p value less than 0.05 was considered statistically significant where: * *p* ≤ 0.05, ** *p* ≤ 0.01, *** *p* ≤ 0.001 and **** *p* ≤ 0.0001

Fibrosis is a prominent pathological feature in skeletal muscle [41–44], and highly correlated with IMAT formation [15–18, 45–48]. To assess any strain and/or sex differences in the fibrotic response post injury, we compared collagen content 21 days after a CTX and GLY injury in Bl6, 129S and CD1 male and female mice (Fig. 3A and Fig. S3), as described previously by a Sirius red staining (% of TA area). Interestingly, we found no sex differences between all three mouse strains after either CTX or GLY injuries (F = M; Fig. 3C). Upon comparing between strains, a CTX injury generally induced comparable amounts of fibrosis in both sexes across the different strains (Bl6 = 129S = CD1; Fig. 3D). However, 129S mice were the most resistant to fibrosis after a GLY injury, compared to Bl6 and CD1s, which had comparable amounts of fibrosis between each other (129S << Bl6/CD1; Fig. 3D). This resistance in fibrotic response in the 129S mice is further appreciated when comparing injury models. While a GLY injury caused a significant fibrotic response in both sexes of Bl6 and CD1 mice compared to a CTX model (GLY >> CTX), 129S mice had comparable fibrotic scaring between injury models (GLY = CTX; Fig. 3E).

Taken together, sex does not influence the amount of fibrotic scarring, however fibrosis is dependent on injury type (GLY >>> CTX) and strain (129S <<< CD1/Bl6; Fig. 3F).

### Myofiber regeneration is strain dependent

**Fig. 4:**
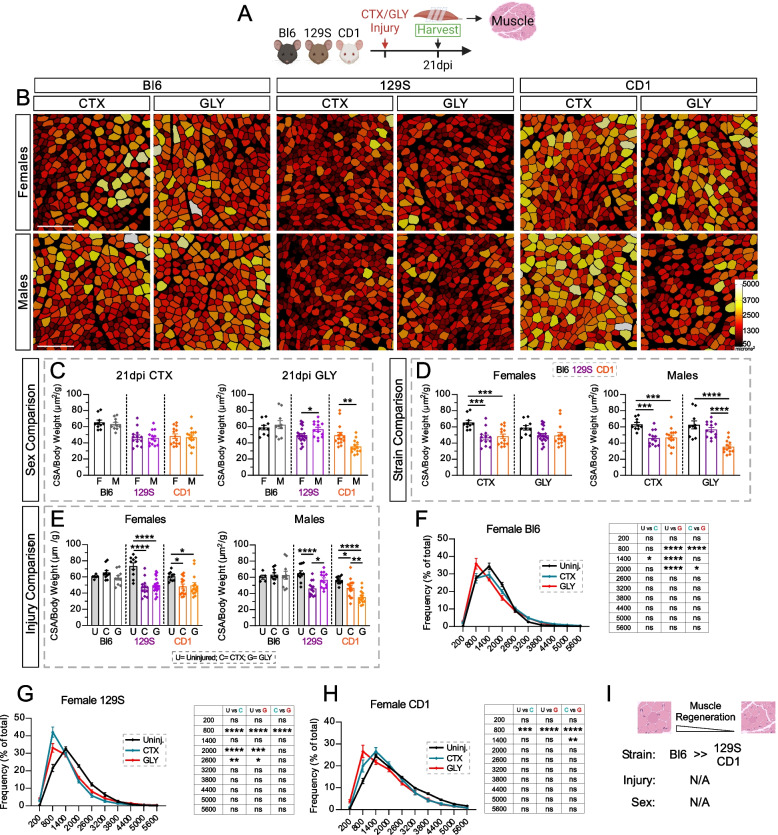
Myofiber regeneration is highly strain, sex, and injury dependent. **A** Experimental outline. **B** Individual muscle fibers, stained by PHALLOIDIN and false color-coded according to cross-sectional area (CSA; µm^2^), 21 days post Cardiotoxin (CTX) or Glycerol (GLY) injury in C57BL/6J (Bl6), 129S1/SvlmJ (129S) & CD1 females (F) and males (M). Scale: 500 µm. **C-E** Average CSA normalized to mouse body weight (µm^2^/g) 21 days post CTX or GLY injury, in both sexes from BL6, 129S & CD1 mice. Data are grouped to compare between: **C** sexes within the same injury and mouse strain; **D** strains within the same sex and injury; **E** injury model within the same sex and strain. **F**–**H** Distribution of myofiber size (µm^2^) as a percentage of total fibers (%) of uninjured muscle, CTX and GLY injured in female: **F** Bl6, (**G**) 129S and (**H**) CD1. **C-H** Uninjured data was obtained from Fig. 1. **I** *Summary data:* the Bl6 strain has the highest regenerative myofiber efficiency. All data are represented as mean ± SEM. An unpaired two-tailed t test or a one-way ANOVA followed by a Dunnet’s multiple comparison was used. A p value less than 0.05 was considered statistically significant where: * *p* ≤ 0.05, ** *p* ≤ 0.01, *** *p* ≤ 0.001 and **** *p* ≤ 0.0001

Murine muscle injury models are widely utilized to define the signaling mechanisms required for successful myofiber regeneration. Multiple studies have focused on comparing the effects of different injury models on muscle regeneration, within the same strain and sex [30, 31]. Here we evaluated differences in myofiber recovery 21 days after two injury models (CTX and GLY), three mouse strains (Bl6, 129S and CD1) and both sexes (Fig. 4A). As a proxy for myofiber regeneration efficiency, we measured the average cross-sectional area (CSA) of regenerated myofibers (Fig. 4B), as previously mentioned above.

Focusing on sex, we found that males tended to display higher CSA compared to females (Supplemental Fig. 4A). In Fig. 1, we found sex-differences in myofiber size pre-injury that could be accounted for by normalizing CSA to body weight. Applying this normalization post injury here as well, all sex differences previously seen after a CTX injury were accounted for (Fig. 4B and C). However, we still detect sex differences in myofiber regeneration only after a GLY injury. For example, 129S males had a higher CSA average, while CD1 males had significantly lower CSA compared to females (Fig. 4C). To note, Supplemental Figs. 2A-C contain the raw un-normalized CSA results.

We next compared differences between strains and found that, when normalizing CSA to body weight, Bl6 mice displayed the highest myofiber recovery ratio after a CTX injury (CTX: Bl6 >>> 129S/CD1; Fig. 4D). In contrast, myofiber regeneration was similarly efficient across all strains after a GLY injury, besides male CD1 mice, which showed delayed myofiber recovery (GLY: Bl6/129S >> CD1).

Lastly, we assessed overall efficiency of the regenerative process by comparing myofiber CSA before (data used from Fig. 1) and after injury. Surprisingly, Bl6 mice fully recover the CSA of their myofibers 21 days after both injuries to pre-injury sizes (Uninjured = Injured; Fig. 4E and S4D). This demonstrates that Bl6 mice have a high regenerative capacity, wherein they fully restore myofiber size to their uninjured state within 3 weeks post injury. We further assessed the regenerative efficiency by evaluating the distribution of fiber size after both injuries compared to an uninjured distribution. We found that both sexes after a CTX injury had a similar fiber distribution to uninjured muscle (Fig. 4F and Fig. S4E), demonstrating its almost absolute regeneration. Surprisingly, in both sexes a GLY injury had a leftward shift in fiber distribution compared to an uninjured muscle, having more of the smaller fibers compared to an uninjured muscle (Fig. 4F and Fig. S4E). Therefore, while the average size of myofibers after a GLY injury is comparable to uninjured muscle (Fig. 4E and Fig. S4D), when analyzing the distribution of.

fiber size, GLY causes a leftward shift in fiber distribution compared to an uninjured muscle. Hence, Bl6 mice almost fully recover from a CTX injury and to a lesser extent from a GLY injury.

In contrast, females and males of both 129S and CD1 mice had significantly lower average CSA and CSA normalized to body weight 21 days post GLY and CTX injury compared to uninjured muscle (Uninjured >> Injured; Fig. 4E and Supplemental Fig. 4C). Upon assessing their fiber distribution, we observed that 129S and CD1 females had a leftward shift after both injuries compared to uninjured distribution (Fig. 4G and H), which was reflected in the average size of fibers. However, where previously there was no difference in average CSA between injury models in female 129S and CD1 mice (Fig. 4E), the fiber distribution between injuries in both strains had differences. In 129S females, a CTX injury had a leftward shift compared to a GLY distribution (Fig. 4G). Opposing this, in female CD1, a GLY injury had a leftward shift in fiber distribution compared to a CTX distribution (Fig. 4H). Both trends in fiber distribution follow that of the average CSA of their opposite sex of the same strain, indicating strain-specific responses to both injuries.

Similar to our previous findings, male CD1s had a decreased average CSA (absolute and normalized to body weight) after a GLY injury compared to CTX (GLY < CTX), indicating that myofiber regeneration is less efficient after a GLY injury compared to CTX (Fig. 4E and Supplemental Fig. 4C). Interestingly, male 129S show the opposite trend, with a higher CSA after a GLY injury compared to CTX (GLY > CTX; Fig. 4E and Supplemental Fig. 4C). This suggests that the efficiency of regeneration between CTX and GLY are strain dependent. Both male CD1 and 129S fiber distributions followed the trends of their averaged fiber size. In male 129S, a CTX had a leftward shift in fiber distribution compared to a GLY injury and uninjured muscle (Supplemental Fig. 4E). While a GLY injury in male CD1s showed a leftward shift in fiber distribution compared to both CTX and uninjured muscle (Supplemental Fig. 4F).

In conclusion, our data demonstrate that the efficiency of myofiber regeneration is strain and sex dependent. Specifically, we find that Bl6 mice are remarkable myofiber regenerators and restore their myofibers within 3 weeks to pre-injury levels (Fig. 4I).

### IMAT and fibrosis are negatively correlated with myofiber regeneration

**Fig. 5:**
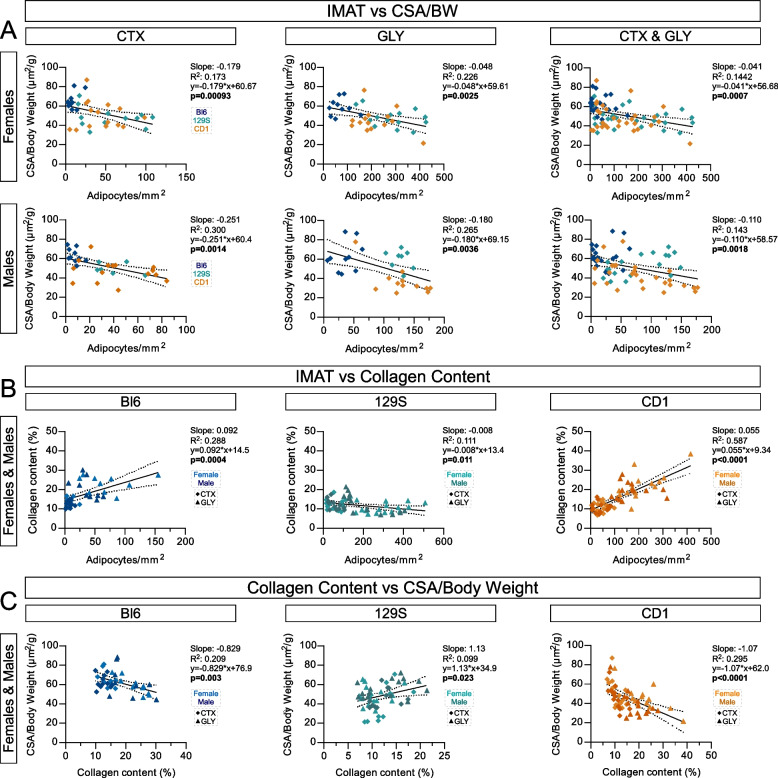
IMAT and fibrosis are positively correlated, both impacting myofiber size. Data for IMAT obtained from Fig. 2, Collagen content from Fig. 3 and average CSA/Body weight from Fig. 4. **A** Correlation between IMAT and CSA/Body weight for every TA, separated by sex and injury model. **B** Correlation between IMAT formation and collagen content separated by strain. **C** Correlation between Collagen content and average CSA/Body weight separated by strain. A Pearson correlation test was used

In humans, IMAT displays a strong inverse correlation to muscle mass and function, most notably within diseases such as muscular dystrophies, diabetes, obesity, spinal cord injuries and sarcopenia [49–52]. Based on our observation that 129S and CD1 form both more IMAT but not fully regenerate their myofibers post injury compared to Bl6 mice, who form little IMAT and fully recover their myofibers, we next investigated whether increased IMAT formation could also be negatively correlated with myofiber regeneration post injury in healthy mice (Fig. 5A). For every individual TA, both the average CSA normalized to body weight (data from Fig. 4) and its respective amount of IMAT (data from Fig. 2) were graphed, and a simple linear regression test was carried out to calculate the correlation coefficient and the significance of this relationship. For each sex, we combined data from all three strains (Bl6, 129S & CD1), first assessing CTX and GLY injuries separately, as well as combined (Fig. 5A). Data separated by strain can be found in Figure S5A. Despite differences in IMAT distribution, in both sexes and in both, separated and combined data, for injury type, we observed a significant negative correlation between IMAT and myofiber size. Thus, the more IMAT formed, the smaller the myofibers are. These results also suggest that the negative relationship between IMAT and myofiber strength observed in chronic human pathologies may also be true during regeneration of healthy muscle tissue.

Our data demonstrate that acute injuries result in increased collagen deposition, indicative of injury-induced fibrosis (Fig. 3). In chronic conditions, IMAT and fibrosis form together [15–18]. To determine whether IMAT and fibrosis also positively correlate with each other post-acute injuries, we graphed the amount of IMAT to its respective amount of fibrosis for each mouse followed by a simple linear regression test. We detected a clear positive correlation between IMAT and fibrosis in Bl6 and CD1 mice especially after a GLY injury (Fig. 5B and S5B). However, 129S animals displayed a slight negative correlation post GLY injury, which is most likely due to them being the most resistant to fibrosis out of all three strains. Thus, our data demonstrate that IMAT and fibrosis are interconnected and arise simultaneously.

Similarly to IMAT, fibrosis also has a strong negative correlation to muscle function in humans [41–43, 53]. Therefore, we also assessed whether increased fibrosis negatively correlated with reduced myofiber size post injury by plotting each animal’s individual fibrosis percentage with its CSA. We observed a clear negative correlation between smaller myofibers and larger amounts of fibrosis in both Bl6 and CD1 mice (Fig. 5C and S5C). As expected, this correlation was more pronounced post GLY injury, which induces more IMAT and fibrosis than CTX. 129S animals displayed a weak positive correlation, which, similar to the correlation to IMAT results, could be due to them being resistant to fibrosis. Together, this indicates that increasing amounts of fatty fibrosis might have a negative impact on myofiber regeneration post injury.

### Backcrossing a Bl6 strain to the 129S strain provides a more suitable mouse model for IMAT pathology

The Bl6 mouse is one of the most widely used strains in skeletal muscle research [29, 54]. However, we find that this strain has a limited amount of IMAT and a high muscle regenerative capacity, therefore failing to best model human pathology affected by IMAT. In stark contrast, the 129S strain has high amounts of IMAT and does not fully recover myofiber size after injury (Fig. 4), indicating that the 129S mouse might represent a better strain to model human IMAT and muscle pathology. To determine how genetically resilient the lean phenotype of Bl6 mice is, we aimed to test two scenarios of backcrossing; one where the starting point is a pure Bl6, crossed with a pure 129S strain, and another where a predominant Bl6 (referred to as Bl6_mix_) is crossed with a pure 129S, modeling the real-life scenario of starting a new genetic cross on a mixed background strain and backcrossing to a pure 129S strain.

**Fig. 6:**
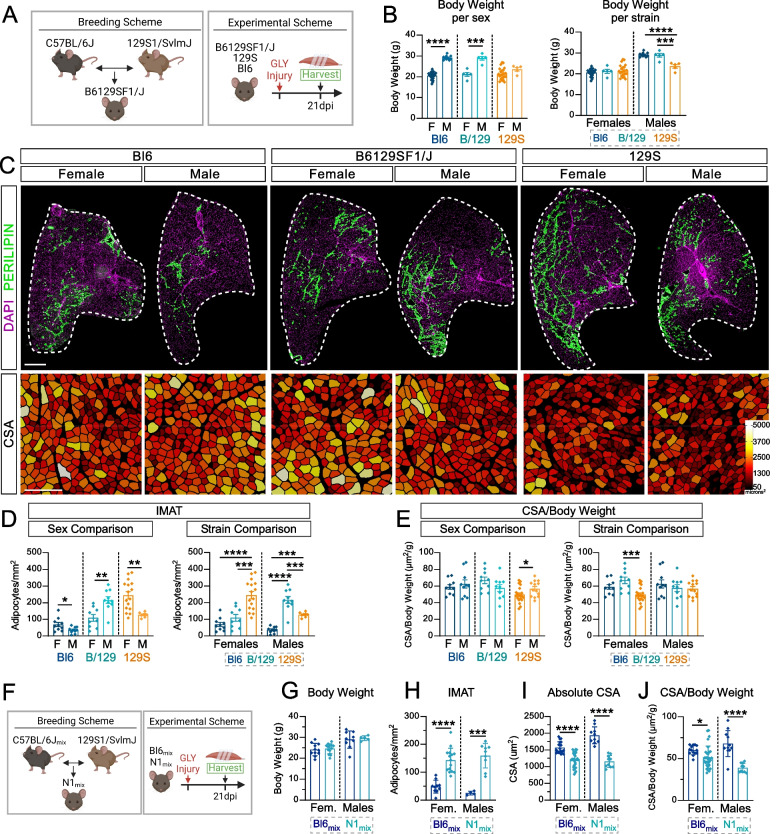
A single cross between C57BL/6J and 129S1/SvlmJ increases IMAT formation providing a superior model for studying IMAT. **A** Breeding and experimental outline. **B** Body weight (g) of adult, 10-week-old C57BL/6J (Bl6), 129S1/SvlmJ and B6129SF1/J (B/129) males and females. Data are group to compare between: *(Left)* sex within the same strain; *(Right)* strains within the same sex. **C** *(Top)* Immunofluorescence of adipocytes (PERILIPIN^+^ cells; green) 21 days post injury (dpi) with Glycerol (GLY) in females (F) and males (M) from Bl6, B/129 & 129S mouse strains. Nuclei were visualized through DAPI (magenta). Scale bar: 500 µm. *(Bottom)* Muscle fibers were visualized through PHALLOIDIN and color-coded according to their cross-sectional area (CSA; um^2^). Scale bar: 250 µm. **D** Quantification of adipocytes normalized to injured area (adipocytes/mm^2^); and (**E**) average CSA normalized to body weight (µm^2^/g) 21 days post GLY injury in Bl6, 129S and B/129 female and male mice. **B**,** D**,** E** Data are grouped to compare between *(Left)* sexes within the same strain; *(Right)* strains within the same sex. **B-E** Data and images for Bl6 and 129S strains were taken from Fig. 1 for body weight, Fig. 2 for IMAT and Fig. 4 for muscle regeneration. **F** Breeding and experimental outline. **G** Body weight (g) of adult 10-week-old mixed C57BL/6J (Bl6_mix_) and mixed N1 progeny (N1_mix_) females and males. **H** Quantification of adipocytes normalized to injured area (adipocytes/mm^2^); **I** average cross-sectional area (CSA; µm^2^); and (**J**) average CSA normalized to body weight (µm^2^/g) 21 days post GLY injury of Bl6_mix_ and N1_mix_ of both sexes. All data are represented as mean ± SEM. An unpaired two-tailed t test or a one-way ANOVA followed by a Dunnet’s multiple comparison was used. A p value less than 0.05 was considered statistically significant where: * *p* ≤ 0.05, ** *p* ≤ 0.01, *** *p* ≤ 0.001 and **** *p* ≤ 0.0001

For the first scenario, we analyzed the hybrid mouse B6129SF1/J (referred to as B/129; Fig. 6A), F1 offsprings derived from crossing a pure C57BL/6J female to a 129S1/SvlmJ male. We assessed IMAT and muscle regeneration 21 days after a GLY injury and compared it against its parental strains (Fig. 6A). Upon tissue harvest, we measured body weights and found that B/129 mice show a sex difference, where males are heavier than females (M > F), as seen in the Bl6 strain (Fig. 6B; data for Bl6 and 129S from Fig. 1). When comparing between strains, we found no difference in body weights for females (Bl6 = B/129 = 129S), however 129S males had the lowest body weight (Bl6/B/129 >> 129S; Fig. 6B). Therefore, the hybrid B/129 mice show a more similar body weight phenotype as the parental Bl6 strain rather than the 129S.

We next assessed IMAT infiltration by quantifying the number of adipocytes (PERILIPIN^+^) per injured area (Fig. 6C) and found that male B/129 mice from more IMAT.

compared to females (M >> F; Fig. 6D). This is in contrast to both parental strains, where we showed that females have more IMAT over males. When comparing between strains, 129S females formed significantly more IMAT than Bl6 and B/129 mice (Bl6 = B/129 << 129S; Fig. 6D), indicating that the B/129 hybrid females are more similar to the Bl6 strain. However, male B/129 mice had the highest IMAT compared to both parental strains (B/129 >> 129S >> Bl6; Fig. 6D), displaying a unique phenotype from its parental strains.

We also evaluated muscle regeneration 21 days after a GLY injury as described above. To account for the impact of body weight on myofiber size, we again normalized body weight to average CSA (raw CSA values can be found in Supplemental Fig. 5A). We found no difference between sexes in the hybrid B/129 and pure Bl6 strains (F = M), while 129S males had larger myofibers compared to females (M > F; Fig. 6E; data for parental strains from Fig. 4). When comparing between strains, female B/129 display larger myofiber CSA compared to 129S but not Bl6 (Bl6 = B/129 >> 129S; Fig. 6E). We found no difference in average CSA amongst the males of all three strains (Fig. 6E), thus, our results highlight that the B/129S hybrids, at least in males, become more fatty, while they retain the high myofiber regeneration capabilities of the Bl6 strain.

For complex mouse genetics experiments, multiple mouse alleles must be intercrossed to achieve experimental mice with the correct number of genetically modified alleles. Often, the different allele-carrying mice are not on the same genetic background, creating mixed backgrounds of varying degrees. We sought to model this scenario by starting with a predominantly Bl6 mouse (~ 75–80% C57BL/6J with the remaining percentage a mix between 129S1/SvlmJ and CD1, referred to as Bl6_mix_) and backcross to a pure 129S1/SvlmJ mouse, giving rise to an N1 generation (referred to as N1_mix_, as the parental Bl6 strain is not pure) (Fig. 6F). We found that both Bl6_mix_ and N1_mix_ mice showed sex differences in body weight, with males weighing more than females (M > F; Supplemental Fig. 5B and C); and no difference in body weight within both sexes when comparing between strains (Bl6_mix_ = N1_mix_; Fig. 6G). We quantified the amount of IMAT 21 days after a GLY injury and found no difference between females and males in both the Bl6_mix_ and N1_mix_ (M = F; Supplemental Fig. 5D). Importantly, both female and male N1_mix_ mice had significantly higher IMAT compared to Bl6_mix_ mice (N1_mix_ >> Bl6_mix_; Fig. 6H), indicating that one backcross on a Bl6_mix_ strain produces higher amounts of IMAT compared to the pure Bl6 strain (Fig. 6D). We also assessed myofiber regeneration 21 days after a GLY injury and found that male Bl6_mix_ had a higher average CSA compared to females (M > F), whereas there was no difference between female and male N1_mix_ mice (M = F; Supplemental Fig. 5E). Upon normalizing average CSA to body weight, we find no differences between male and female Bl6_mix_ mice, while male N1_mix_ mice had a lower CSA/Body weight ratio (Supplemental Fig. 5F). When comparing between strains, both sexes of N1_mix_ mice had a significantly lower average CSA and CSA/body weight ratio compared to Bl6_mix_ (N1_mix_ << Bl6_mix_; Fig. 6I and J), suggesting that crossing the 129S strain to a Bl6_mix_ has a significant impact on myofiber regeneration that better recapitulates human disease. Taken together, a few simple backcrosses to a 129S mouse strain allows more IMAT infiltration, thereby better recapitulating human pathology.

## Discussion

Skeletal muscle is an important organ [55] affected by many diseases that cause a decrease in muscle mass and muscle quality, leading to increased morbidity and mortality [1, 4, 12, 56]. In the present study, three commonly used mouse strains were evaluated for their suitability for modeling pathological human features such as intramuscular fat (IMAT), fibrosis and myofiber damage. We included both isogenic, Bl6 and 129S, as well as outbred CD1 strains due to their opposing genetic characteristics and uses. Due to their identical genetical makeup, isogenic strains have the advantage of studying the physiological effects of specific genes of interest, whereas outbred strains with their high genetic variability better model the genetic diversity of the human population. We investigated how variables such as sex, injury model, and genetic backcrossing affected these pathological features. We found that Bl6 mice are extremely lean and have a high efficiency to regenerate damaged myofibers. In contrast, both 129S and CD1 strains display high IMAT infiltration and reduced myofiber regeneration. Additionally, the 129S strain was the most resistant to injury-induced fibrosis compared to Bl6 and CD1 strains. Importantly, variables such as sex and injury model heavily influence phenotypes. For example, females have higher IMAT formation compared to males after a Glycerol (GLY) injury, and GLY in turn not only induces more IMAT, but also elicits a higher fibrotic response compared to a Cardiotoxin (CTX) injury. Thus, careful considerations have to be made for which strain and sex to use when modeling human pathology. In fact, our data highlight that, surprisingly, Bl6 mice, especially males, appear to be the worst choice to study IMAT formation.

### Considerations when studying IMAT

There is strong evidence indicating that mouse strains differ in their muscle physiology [57–61], due to the genetic variability between them [62]. Which strain to select depends largely on the area of research within skeletal muscle and remains a difficult task to undertake. Backcrossing to a desired strain is time consuming, as well as financially costly. Additionally, the molecular and genetic differences between all strains are not completely resolved, preventing any possible prediction on which strain will best serve one’s study. Here, we hypothesize that, when studying IMAT a high adipogenic predisposition may allow detectable changes in IMAT. For instance, if a gene of interest or a pharmacologic intervention represses IMAT, if studied on a Bl6 strain, it is possible that no difference will be found, or the intervention must be strong enough to induce a difference in the IMAT-resistant strain. Conversely, if studied on a 129S or CD1 strain, repression of IMAT can be more readily noticed. In our experience, even if the outcome hypothesized is an increase in IMAT, Bl6 are still extremely resistant to IMAT. Additionally, we have recently shown that the amount of IMAT can be further increased in the 129S strain by turning off the Hedgehog pathway [35]. Therefore, if studying IMAT, both 129S and CD1 strains are recommended.

We and others have found that IMAT is extremely limited during muscle homeostasis and, therefore, requires an injury to induce its formation [32, 34, 35, 38, 39, 63, 64]. While both CTX and GLY injury models induce IMAT formation, we found that a GLY model causes roughly 4–fivefold more IMAT compared to a CTX injury across all strains. Interestingly, the amount of IMAT is sex and injury dependent. Females of all strains had a higher adipogenic predisposition compared to males after a GLY injury, while no sex difference was observed in IMAT after a CTX injury. These results were further observed and supported by Pisani et al. where females of the hybrid mouse strain B6D2, a cross between C57BL/6 and DBA/2 strains, had significantly more IMAT than males after a GLY injury at 12 months of age [65]. Fearing et al. also show an age-dependent phenotype where older C57BL/6J females have higher amounts of IMAT compared to males following a CTX injury [66]. Additionally, in a C57BL/6J strain, McHale et al. found that females had higher amount of IMAT 7- and 14 days post CTX injury, but 21 days after injury, both sexes had comparable amounts [67]. Thus, while a GLY injury causes more IMAT to form, there are clear sex differences for IMAT formation, and should be taken in consideration in the study design.

Another aspect of IMAT to consider is its cellular composition and thereby potential metabolic impact. IMAT, considered to be white adipose tissue (WAT), can also contain brown adipose tissue (BAT). BAT displays strong metabolic activity in that it consumes lipids for heat production, as opposed to WAT that is primarily being used for lipid storage. Interestingly, recent work demonstrated that the conversion from WAT to BAT depended on the mouse strain. Gorski et al. found differences in BAT formation in vitro between 129S6/SvEvTac and C57BL/6J mouse strains, indicating possible in vivo differences in metabolic responses [68]. Furthermore, they also show that isolated pre-adipocytes from 129S6/SvEvTac females displayed higher BAT formation in vitro compared to males, indicating additional conserved sex differences in BAT response [68]. Pointing to a potential protective mechanism of this WAT to BAT conversion, Almind et al., found that a high fat diet induced BAT in muscles of 129S mice compared to Bl6 mice [69]. Since BAT can promote muscle regeneration [70], further experiments investigating the effects on the conversion of white IMAT into brown IMAT would have potential implications for promoting muscle health. It also highlights the importance of strain selection for modeling human conditions in which BAT vs WAT are affected.

### Relevance of mice as model to study human pathology

It remains difficult to assess which mouse strain is a better translational model for human pathology for multiple reasons. First, mice are extremely lean, and some form of exogenous perturbation is required to induce IMAT. Despite being a commonly used tool in the field, injectable muscle injuries are an artificial stimulus and do not directly model the human condition. They are, however, useful tools to study the cellular and molecular mechanism on why and how IMAT forms. Secondly, phenotypic similarities in IMAT do not always predict better modeling of human pathology. For example, both CD1 and 129S strains show high IMAT formation following injury but have contrasting fibrotic responses. Stating that one of these strains better recapitulates human pathology is challenging as the causal relationship between human pathological IMAT and fibrosis is unknown. Therefore, it is unclear which strain, if any, would recapitulate human physiology best. Another consideration is how a certain human pathology is being induced in mice. For example, rotator cuff tears in humans lead to a significant replacement of skeletal muscle with IMAT. However, the suprascapular nerve (SSN) is only rarely damaged. In contrast, murine rotator cuff injury models are induced by tenotomy and neurotomy of the SSN nerve, which will result in IMAT infiltration but also denervation of the muscle (reviewed in [71, 72]). Thus, murine injury models may not faithfully recapitulate the human condition and caution is advised when comparing phenotypes.

### Variability in fibrotic response

In most pathological conditions or injuries, such as a rotator cuff tear, fibrotic scar tissue is a predominant feature in humans [41–44] and can be modeled in the mouse [26, 73–75]. However, a comparison of fibrotic response between different wild-type strains, injury models and sex are mostly lacking. Here, we found that a GLY injury induces a higher fibrotic response compared to a CTX model, while the 129S strain is the most resistant to injury-induced fibrosis.

Looking at any potential correlation between fibrosis and myofiber size, we also found that the higher the collagen content the smaller the myofibers are. However, this response was highly strain dependent as only Bl6 and CD1 but not 129S mice displayed a strong negative correlation. These results fit with previous studies that have shown a negative impact of fibrosis on myofiber regeneration post-acute injuries [76–78].

In humans, fibrosis and IMAT form simultaneously [16, 45–48, 53, 79, 80]. This leads to the question of whether both form in response to a shared injury cue or are controlled independently. While not directly answering this question, we found a tight correlation between IMAT and fibrosis in that an increase in IMAT does predict an increase in fibrosis and vice versa. This positive correlation between IMAT and fibrosis is the most prevalent post GLY injury, which, fittingly, also causes the most IMAT and fibrosis. Interestingly, this correlation was strain dependent and limited to Bl6 and CD1 mice similarly to the negative correlation between fibrosis and myofiber size. Taken together, both Bl6 and CD1 strains are susceptible to injury-induced fibrosis, GLY causes a stronger fibrotic response compared to a CTX model, IMAT formation does predict a fibrotic response, and fibrosis is negatively correlated with myofiber regeneration but in a strain-dependent fashion.

### Strain selection for preclinical studies in skeletal muscle regeneration

Despite mice being extremely useful for understanding and developing drugs to treat human conditions, there is a substantial challenge when it comes to translating these findings to human trials [81–85]. Even though there are differences between mice and human species, aspects in study design such as sample size, sex inclusion and blinding, amongst others, are extremely important to effectively bring findings in the mouse as a therapeutic intervention to the human population [81]. Another important variable to adequately mimic human pathology is the mouse strain and its genetic background. Several studies have reported that muscle regeneration is dependent on the genetic background [86, 87]. Bl6 mice are considered the standard mouse strain and have been extensively utilized in studies focused on skeletal muscle [29, 54]. Our results demonstrate that Bl6 fully regenerate their myofibers 21 days post CTX and GLY injury, highlighting the high regenerative capacity this strain has. Furthermore, supporting our data, Hardy et. al found that myofiber diameter further increased one month after a CTX injury in Bl6 mice [30]. In comparison, both 129S and CD1 strains failed to fully regenerate muscle fibers to pre-injury size 21 days after injury, indicating that these strains have a delayed or less efficient muscle regeneration.

Strain selection can heavily impact the phenotype in a disease model. As mentioned previously, before the identification of the DBA/2J mouse as a superior strain to study DMD [26–28], one major hurdle in the field was the lack of skeletal muscle degeneration and many efforts were made to address this [85]. One study identified the extreme capability of muscle stem cells to regenerate in the mdx model on a C57BL/6J background leading to mild muscle defects [88]. They were able to reduce the proliferative ability of muscle stem cells by shortening the telomeres within these stem cells, leading to less efficient muscle regeneration, and therefore creating a more desirable muscle degeneration phenotype [88]. Our data further support the notion that the Bl6 strain has the highest muscle regenerative capacity compared to 129S and CD1 strains. Therefore, the Bl6 strain is a suitable model to study complete myofiber regeneration. In contrast, both 129S and CD1 strains do not recover their myofibers as efficiently 21 days post injury. These results reflect subtle strain-specific differences in post regenerative myogenesis, which might cause different outcomes in preclinical studies. It is important to point out that our conclusions are specific to the injury models that we have chosen. For example, we show that 129S mice do not fully regenerate their muscle after both CTX and GLY injuries 21 days post injury. However this strain is resistant to muscle atrophy induced by hindlimb suspension compared to NOD/ShiLtK, NZO/HILtJ and A/J strains [59]. Therefore, assumptions cannot be made of potential phenotypes in other types of injury models or disease models not included in this study.

### The relationship between IMAT and muscle regeneration

IMAT is a shared hallmark of many human conditions that are impacted by defective muscle regeneration. Despite the strong negative correlation, however, it remains unclear whether IMAT is the cause or consequence of failed regeneration. Therefore, determining the effects of IMAT on skeletal muscle has been a long-standing question. Factors that hinder answering this question in preclinical mouse models are the confounding impact of the disease itself, and arguably the selection of mouse strain, mostly performed on a Bl6 background, which are resistant to IMAT formation. IMAT has been shown to have a negative correlation with muscle strength [18, 61], indicating IMAT either directly impacts myofibers causing a decrease in force or IMAT hinders muscle contraction as a non-contractile tissue within a contractile tissue [89]. While not directly testing this hypothesis, we found a strong negative correlation between IMAT and myofiber size after injury. This suggests the interesting possibility that, without the added variable of disease, IMAT could possibly have a negative impact on myofiber regeneration, thereby impacting muscle strength and function. Further in-depth studies are required to understand how IMAT impacts muscle regeneration.

### Practical implications and effects of background strain on IMAT and muscle regeneration

For a mouse to be considered on a pure strain, 10 + generations of backcrosses to the desired strain are required. That amounts to 99.9% genetic similarity to the backcrossed strain, and 2.5–3 years to achieve. Because this is, in most cases, unattainable, a more approachable breeding plan is to backcross for 5 generations, obtaining 94% of genetic similarity, which requires 1.5 years. However, strain-specific traits might manifest earlier. Chan et al. assessed the number of required crosses from a Bl6 to a 129S strain until desired differences of both strains were observed, such as susceptibility to stress, high cognitive performance, and strain-dependent hypothalamus–pituitary–adrenal (HPA) stress axis responses. They concluded that three generations of crossing to a 129S strain was sufficient to obtain the desired traits [90]. As our results demonstrated that both 129S and CD1 strains are superior models for IMAT than the Bl6 strain, we also asked how genetically resilient the IMAT resistance is in Bl6 mice. For this, we assessed the phenotype of the hybrid mouse B6129SF1/J (B/129), which are F1 progeny between pure Bl6 and 129S mice. In the field of neurobiology, Bl6 and 129S strains have distinct behavior and neurobiological differences [90–94]. For example, Hansen et al. assessed the B6129SF1/J hybrid mouse and found that it presents distinct features from its parental Bl6 strain, such as being more sensitive to stress, while the parental Bl6 strain has a higher locomotor activity and exploratory behavior [94]. In the present study, we find that this hybrid strain resembles both strains as well as having unique phenotypes that differ from its parental strains. For instance, female B/129 mice have comparable amounts of IMAT compared to the Bl6 strain, while male B/129 mice have comparable average CSA to both strains 21 days post GLY injury. Uniquely, B/129 males have the highest IMAT compared to both parental strains. Therefore, one cross between Bl6 and 129S strains is sufficient in males to obtain the desired IMAT phenotype as seen in the 129S strain. We also emulated a real-life scenario where the starting point of a cross is a mixed, but predominantly, Bl6 background. Parting from a mixed Bl6 background, one cross with a pure 129S strain had a significant impact on IMAT and muscle regeneration on their progeny. This indicates that, while outcrossing to another strain is time-consuming and financially costly, depending on the purity of the Bl6 strain, one cross may be sufficient to obtain a more suitable IMAT phenotype.

Importantly, these findings also indicate the existence of genetic modifiers that confer predisposition to IMAT. There is ample evidence for genetic modifiers and their potent impact on the *mdx* mouse model. For example, considering fibrosis, *mdx* mice on a DBA/2J instead of the common Bl6/Bl0 background displayed a more prominent fibrotic phenotype, faster muscular degeneration, and shorter lifespan, thereby resembling human pathology more accurately [26–28, 95, 96]. Investigating the difference in the fibrotic phenotype led to the discovery of the genetic modifier TGF-beta-binding protein 4 gene (*Ltbp4*) as the cause of increased fibrosis, which is specifically present in the DBA/2J mouse strain [28, 86, 96–98]. Similarly, other genetic modifiers that impact the disease severity of DMD have been identified such as Osteopontin [99] and Annexin 6A [100]. Therefore, identifying genetic modifiers responsible for IMAT formation would allow for building better mouse models but may also enable uncovering novel genetic predispositions to IMAT in humans.

### Sex as an important variable in preclinical studies focused on skeletal muscle

Not only is there evidence indicating sex differences in skeletal muscle physiology [101], but it has been nearly a decade since the National Institutes of Health (NIH) has been actively working towards inclusion of both sexes in preclinical research [102]; however disparities still exist [103, 104]. Studies tackling differences due to sex, strain and injury type have mainly focused on one or two of these variables. For instance, it has previously been reported that 129S mice have higher IMAT following a GLY injury compared to Bl6 [105]. However, that study only focused on males. Interestingly, females of the hybrid mouse strain B6D2, a cross between C57BL/6 and DBA/2 strains, had significantly more IMAT than males after a GLY injury at 12 months of age [65]. Female Bl6 mice also display exacerbated IMAT infiltration of the sarcopenic rotator cuff muscles [74]. There is also evidence indicating that females have lower average CSA and comparable IMAT compared to males in a C57BL/6J strain 21 days post CTX [67]. In contrast, rotator cuff tenotomy-induced atrophy was exacerbated in male compared to female Bl6 mice [106]. Therefore, we considered comparing both sexes an important aspect of this study, as a comparison between sexes of different strains and across multiple injuries are not available. In our study, we found sex-dependent and independent outcomes that are additionally influenced by strain and injury type. For instance, sex differences in body weight are strain dependent. Body weight of both Bl6 and CD1 show sex differences with males being heavier than females, while the 129S strain had no sex differences. This difference in body weight may also affect myofiber size and therefore, needs to be considered when evaluating myofiber size by analyzing and comparing separately. We have shown that this difference can be accounted for when normalizing the average CSA to the body weight of the individual. Interestingly, we find an injury dependent sex difference in IMAT formation. While a CTX injury shows no sex difference in IMAT formation, females have substantially higher IMAT infiltration following a GLY injury than males. Contrastingly, we find no sex differences in fibrosis across all strains and both injuries, indicating that fibrosis is not affected by sex. Intriguingly, myofiber regeneration has sex differences, albeit these differences are injury dependent. For instance, both Bl6 and CD1 strains show a sex difference in myofiber regeneration after a CTX injury. However, after a GLY injury, both Bl6 and 129S show a sex difference in myofiber size. Therefore, careful analysis should be taken when analyzing myofiber regeneration in both sexes. We believe that understanding sex-dependent variables in the context of muscle regeneration and health are fundamental to improving preclinical studies and to aid in further research that focus on ameliorating disease progression of both sexes.

### Concluding remarks

Taken together, our study shows the importance that strain selection can have on studies involving IMAT, muscle regeneration and fibrosis and how these are affected by sex and injury models. Through a comprehensive examination of three commonly utilized mouse strains, we have delineated distinct phenotypic differences in intramuscular fat deposition, fibrotic response, and muscle regeneration post-injury. Notably, Bl6 mice demonstrate robust myogenic regenerative capabilities, high fibrotic response and restricted IMAT formation. Conversely, 129S and CD1 strains exhibit heightened susceptibility to intramuscular fat accumulation, as well as reduced myofiber regeneration. However, CD1 mice show a high fibrotic response while the 129S strain is resistant to injury-induced fibrosis. Furthermore, we show a negative correlation between myofiber regeneration and fatty fibrosis, indicating that IMAT and fibrosis may play negative roles during this process. Our study contributes valuable insights into the complex interplay of genetic and physiological factors shaping skeletal muscle health and emphasizes the importance of strain-dependent phenotypes and sex-specific responses in preclinical models.

## Methods

### Animal studies and muscle injuries

All mice used in this study were mature and between 10–12-weeks old. Male and female 129S1/SvlmJ mice were purchased from Jackson Laboratory (stock #002448), CD1 mice were purchased from Charles Rivers (strain code #022) and B6129SF1/J hybrid (N1F1) mice were purchased from Jackson Laboratory (stock #101,043). Male and female C57BL/6J mice were purchased from the University of Florida’s breeding facilities, obtained from The Jackson Laboratories (stock #000664) and bred in-house; with breeding stock replenished every 5th generation. The mixed Bl6 mice were 70–80% pure C57BL/6J, with the remaining percentage a mix between CD1 and 129S1/SvlmJ. These were crossed with a pure 129S1/SvlmJ mouse (Jackson Laboratory stock #002448) to generate the N1_mix_ mice. Mice were housed in standard ventilated cages at controlled temperature on a 12 h light/dark cycle with ad libitum access to food and water. Mice were under the care of the Animal Care Services at the University of Florida (ACS). All animal work was approved by the Institutional Animal Care and Use Committee (IACUC) of the University of Florida.

For muscle injuries, mice were kept under anesthesia with 2% isoflurane while the Tibialis Anterior (TA) muscles were injected with 30-50uL of 10 nM of Cardiotoxin (CTX; Lotaxan; *Naja pallidum*, L8102-1MG), or with 30-50uL of 50% Glycerol (GLY; Acros Organics, 56–81-5). Injured TAs were harvested 21 days post injury. For uninjured muscles, TAs were harvested from adult 10–12 week-old mice.

### Immunofluorescence and histology

Tissue processing, immunostaining and image acquisition were performed as previously described [34, 35]. Briefly, TA muscles were fixed in cold Paraformaldehyde (PFA) for 2.5 h and cryopreserved overnight in 30% sucrose. For each TA, between 3–4 sections at 10 µm thickness from the mid-belly were obtained with a Leica cryostat and stored at -80 °C. For immunofluorescence staining, slides were blocked with 5% donkey serum in PBS with 0.3% Triton X-100 for 1-2 h at room temperature and primary antibodies were incubated at 4 °C overnight in blocking solution. Primary antibodies used were rabbit anti-Perilipin (1:1000; Cell Signaling, 9349S). Nuclei were visualized through DAPI (Invitrogen, D1306). Secondaries and direct conjugated antibodies were incubated for 45 min at room temperature. These include Alexa Fluor 488 donkey anti-rabbit (1:1000, A-21206) and direct conjugate dye Phalloidin-Alexa 568 (1:250, Molecular Probes # A12380). Finally, slides were mounted (SouthernBiotech; 0100–01) and allowed at least 2 h to dry before imaging. Visualization of collagen deposition was done through the histological stain Sirius Red and analyzed through the Color-Threshold function in ImageJ software.

### Image analysis

All immunofluorescent and histological images were acquired with a Leica DMi8 microscope equipped with a high-resolution color camera. All images were analyzed with Fiji/ImageJ software (1.53c; Java 1.8.0_172). For IMAT quantification, the whole TA cross section was imaged using the Navigator (tile scanning) function. In ImageJ, adipocytes (PERILIPIN^+^ cells) were manually counted using the cell counter plugin and normalized to injured area (mm^2^), identified as fibers with centrally located nuclei, giving the density of IMAT as adipocytes/mm^2^. Average size of adipocytes (µm^2^) was calculated by first obtaining 5–6 areas imaged with a 20 × objective, and then manually measuring the area of adipocytes using ImageJ software. Determining the average cross-sectional area of myofibers was carried out by visualizing fibers through a PHALLOIDIN stain. Uninjured areas, identified as areas that lack centrally located nuclei, were excluded. Myofibers were identified and segmented through Cellpose [107] and processed through our LabelToRois ImageJ plug-in to obtain measurements of the individual fibers and the average was calculated for all fibers [33]. Collagen deposition was calculated by the Color-Threshold function in ImageJ and measured as a percentage of total TA area.

### Statistical analysis

TAs with less than 50% injury were excluded from the analysis. All data were graphed, and statistical analysis run in Graphpad Prism (version 9). All data are shown as ± SEM. When comparing between two groups with one variable, an unpaired two-tailed t-test was used. For more than two groups with one variable, a one-way ANOVA followed by a Dunnet’s multiple comparison test was used. To test the correlation between two related groups, a Pearson’s correlation analysis was used. A p value less than 0.05 was considered statistically significant and the magnitude of significance was denoted as: * < 0.05, ** < 0.01, *** < 0.001 and **** < 0.0001.

### Supplementary Information

Supplementary Material 1.

## Data Availability

No datasets were generated or analysed during the current study.
