# Supplementary material for: Studying intramuscular fat deposition and muscle regeneration: insights from a comparative analysis of mouse strains, injury models, and sex differences
Source: Skelet Muscle. 2024 May 29;14:12. doi: 10.1186/s13395-024-00344-4 (PMC11134715; doi:10.1186/s13395-024-00344-4)
Supplement: Supplementary file 1 — Supplementary Material 1. [file 13395_2024_344_MOESM1_ESM.pdf]

Supplemental Figures

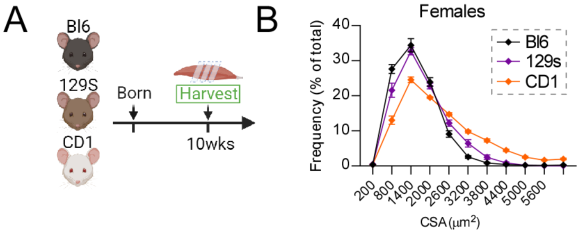

	U vs C	U vs G	C vs G
200	ns	ns	ns
800	****	****	****
1400	ns	****	****
2000	ns	***	**
2600	*	****	*
3200	**	****	**
3800	ns	****	****
4400	ns	**	**
5000	ns	ns	ns
5600	ns	ns	ns

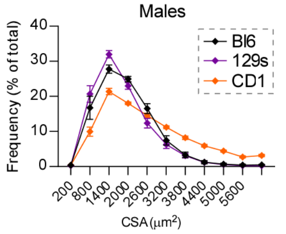

	U vs C	U vs G	C vs G
200	ns	ns	ns
800	*	****	****
1400	**	****	****
2000	ns	****	***
2600	**	ns	ns
3200	ns	*	***
3800	ns	**	***
4400	ns	**	***
5000	ns	*	**
5600	ns	ns	ns

Supplemental Figure 1. Distribution of myofiber size between strains of uninjured muscle. A) Experimental outline. **B)** Histogram distribution of myofiber cross-sectional area (CSA) of uninjured muscle, as a percentage of total fibers (%) of female (*left*) and male (*right*) C57BL/6J, 129S1/SvlmJ & CD1 mice. All data are represented as mean ± SEM. *Bottom*: A two-way ANOVA followed by a Tukey's multiple comparison test was used and statistical significance shown. A p value less than 0.05 was considered statistically significant where: * p≤ 0.05, ** p≤ 0.01, *** p≤ 0.001 and **** p≤ 0.0001.

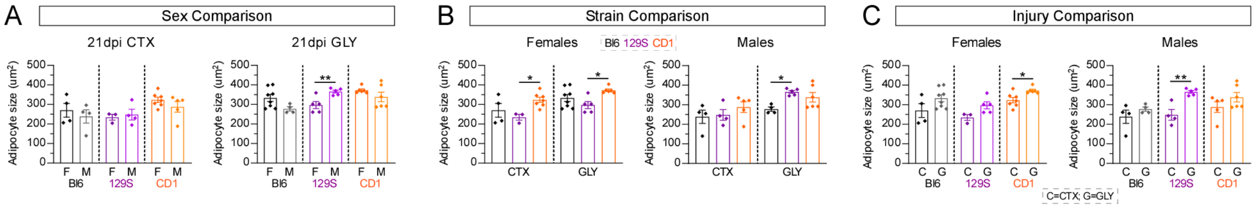

Supplemental Figure 2. Adipocyte size 21 days after injury. Average size of adipocytes (µm^2) 21 days after CTX or GLY injury in male and female C57BL/6J, 129S1/SvlmJ & CD1 mice. Data are grouped to compare between: **A)** sexes within the same strain and injury; **B)** strains within the same injury and sex; **C)** injury models within the same sex and strain. All data are represented as mean ± SEM. An unpaired two-tailed t test or a one-way ANOVA followed by a Dunnet's multiple comparison was used. A p value less than 0.05 was considered statistically significant where: * p≤ 0.05 and ** p≤ 0.01.

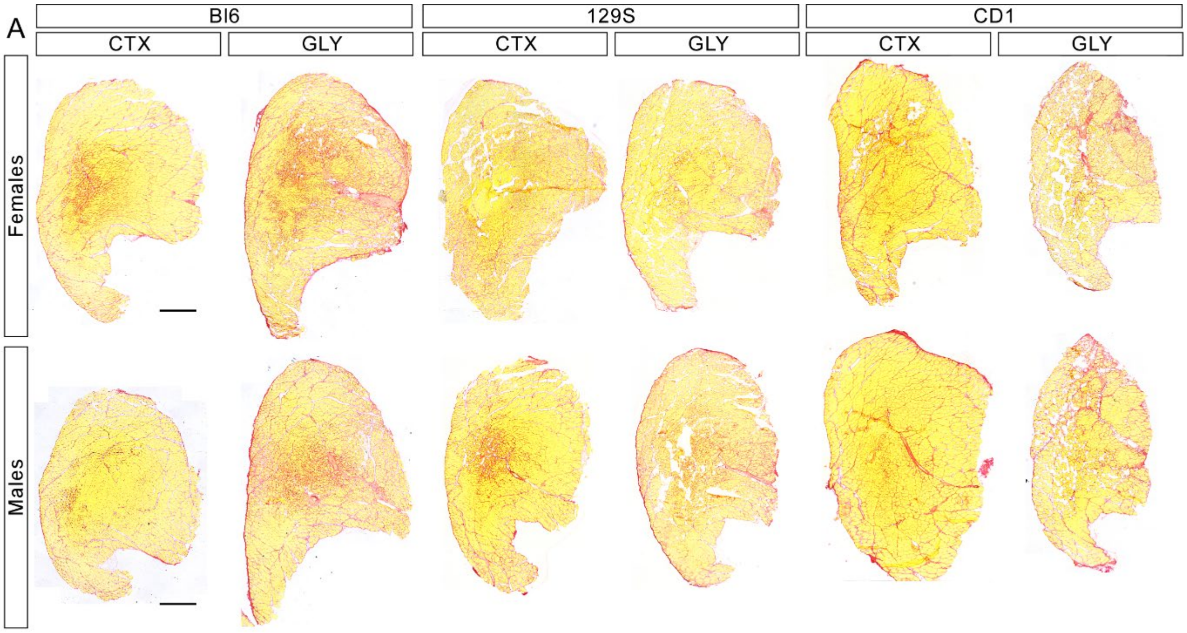

Supplemental Figure 3. Collagen deposition 21 days post injury. A) Cross sections of Tibialis Anterior muscles stained with Sirius Red 21 days post Cardiotoxin (CTX) or Glycerol (GLY) injuries in C57BL/6J, 129S1/Svlmj and CD1 females and males. Scale bar: 500 µm.

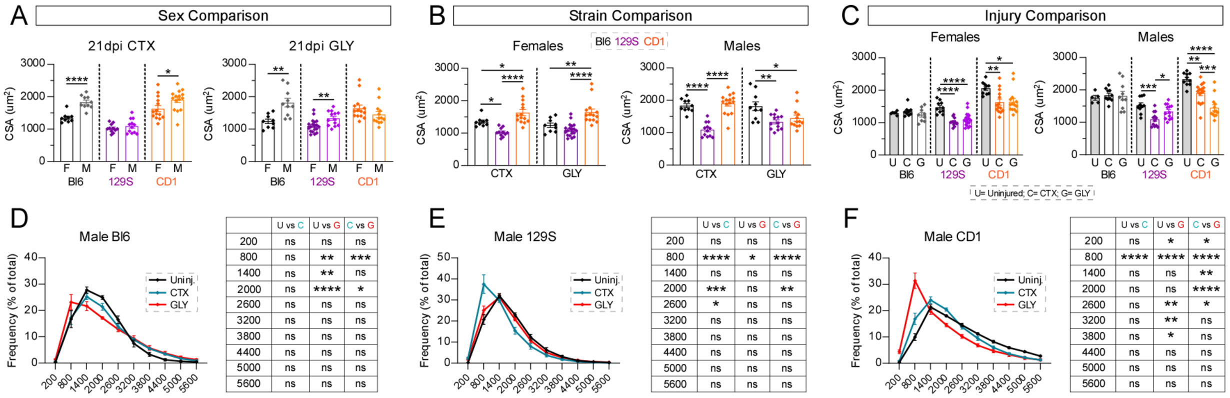

D) Male Bl6

	U vs C	U vs G	C vs G
200	ns	ns	ns
800	ns	**	***
1400	ns	**	ns
2000	ns	****	*
2600	ns	ns	ns
3200	ns	ns	ns
3800	ns	ns	ns
4400	ns	ns	ns
5000	ns	ns	ns
5600	ns	ns	ns

E) Male 129S

	U vs C	U vs G	C vs G
200	ns	ns	ns
800	****	*	****
1400	ns	ns	ns
2000	***	ns	**
2600	*	ns	ns
3200	ns	ns	ns
3800	ns	ns	ns
4400	ns	ns	ns
5000	ns	ns	ns
5600	ns	ns	ns

F) Male CD1

	U vs C	U vs G	C vs G
200	ns	*	*
800	****	****	****
1400	ns	ns	**
2000	ns	ns	****
2600	ns	**	*
3200	ns	**	ns
3800	ns	*	ns
4400	ns	ns	ns
5000	ns	ns	ns
5600	ns	ns	ns

Supplemental Figure 4. Myofiber regeneration between strains 21 days post injury. Average CSA (µm^2) of myofibers 21 days post CTX or GLY injury, in both sexes from C57BL/6J (Bl6), 129S1/SvlmJ (129S) & CD1 mice. Data are grouped to compare between: **A)** sexes within the same injury and mouse strain; **B)** strains within the same sex and injury; **C)** injury within the same sex and strain. **(D-F)** Distribution of myofiber cross-sectional area (CSA) as a percentage of total fibers (%) 21 days after a Cardiotoxin (CTX), Glycerol (GLY) and uninjured muscle in male **D)** Bl6; **E)** 129S and **F)** CD1 strains. **(C-F)** Uninjured data was obtained from Figure 1. All data are represented as mean ± SEM. An unpaired two-tailed t test, a one-way ANOVA followed by a Dunnet's multiple comparison, or a two-way ANOVA followed by a Tukey's multiple comparison test was used and statistical significance shown in table. A p value less than 0.05 was considered statistically significant where: * p≤ 0.05, ** p≤ 0.01, *** p≤ 0.001 and **** p≤ 0.0001.

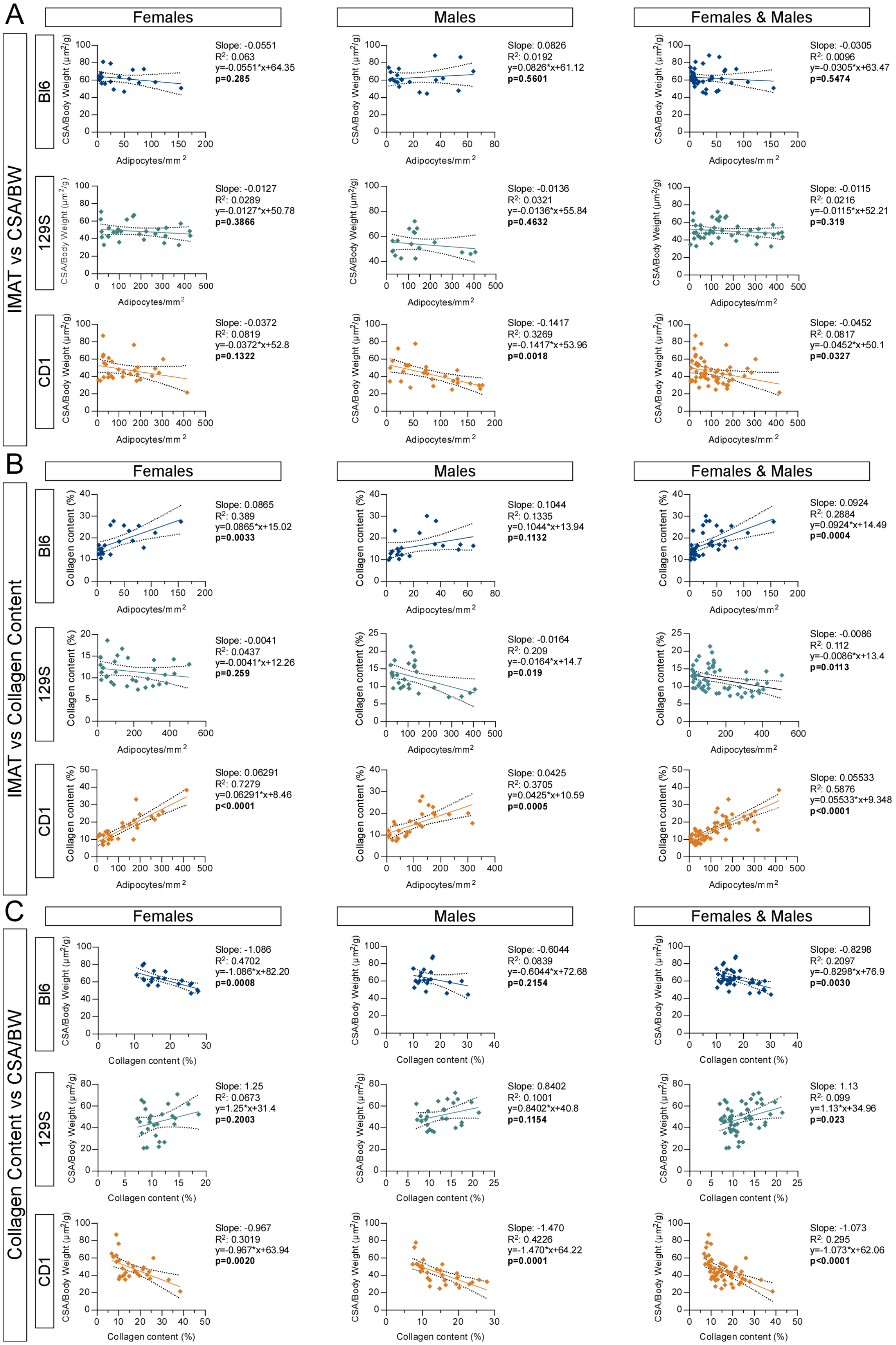
A
B
C
Females
Males
Females & Males
IMAT vs CSA/BW
IMAT vs Collagen Content
Collagen Content vs CSA/BW
Bl6
129S
CD1
CSA/Body Weight (μm²/g)
Collagen content (%)
Adipocytes/mm²
Slope: -0.0551
R²: 0.063
y=-0.0551*x+64.35
p=0.285
Slope: 0.0826
R²: 0.0192
y=0.0826*x+61.12
p=0.5601
Slope: -0.0305
R²: 0.0096
y=-0.0305*x+63.47
p=0.5474
Slope: -0.0127
R²: 0.0289
y=-0.0127*x+50.78
p=0.3866
Slope: -0.0136
R²: 0.0321
y=-0.0136*x+55.84
p=0.4632
Slope: -0.0115
R²: 0.0216
y=-0.0115*x+52.21
p=0.319
Slope: -0.0372
R²: 0.0819
y=-0.0372*x+52.8
p=0.1322
Slope: -0.1417
R²: 0.3269
y=-0.1417*x+53.96
p=0.0018
Slope: -0.0452
R²: 0.0817
y=-0.0452*x+50.1
p=0.0327
Slope: 0.0865
R²: 0.389
y=0.0865*x+15.02
p=0.0033
Slope: 0.1044
R²: 0.1335
y=0.1044*x+13.94
p=0.1132
Slope: 0.0924
R²: 0.2884
y=0.0924*x+14.49
p=0.0004
Slope: -0.0041
R²: 0.0437
y=-0.0041*x+12.26
p=0.259
Slope: -0.0164
R²: 0.209
y=-0.0164*x+14.7
p=0.019
Slope: -0.0086
R²: 0.112
y=-0.0086*x+13.4
p=0.0113
Slope: 0.06291
R²: 0.7279
y=0.06291*x+8.46
p<0.0001
Slope: 0.0425
R²: 0.3705
y=0.0425*x+10.59
p=0.0005
Slope: 0.05533
R²: 0.5876
y=0.05533*x+9.348
p<0.0001
Slope: -1.086
R²: 0.4702
y=-1.086*x+82.20
p=0.0008
Slope: -0.6044
R²: 0.0839
y=-0.6044*x+72.68
p=0.2154
Slope: -0.8298
R²: 0.2097
y=-0.8298*x+76.9
p=0.0030
Slope: 1.25
R²: 0.0673
y=1.25*x+31.4
p=0.2003
Slope: 0.8402
R²: 0.1001
y=0.8402*x+40.8
p=0.1154
Slope: 1.13
R²: 0.099
y=1.13*x+34.96
p=0.023
Slope: -0.967
R²: 0.3019
y=-0.967*x+63.94
p=0.0020
Slope: -1.470
R²: 0.4226
y=-1.470*x+64.22
p=0.0001
Slope: -1.073
R²: 0.295
y=-1.073*x+62.06
p<0.0001

Supplemental Figure 5. Correlations between IMAT, fibrosis and myofiber size. Correlations separated by sex and strain 21 days post Cardiotoxin and Glycerol combined between: **A)** average cross-sectional area normalized to body weight ($\mu m^2/g$) to IMAT formation ($adipocytes/mm^2$); **B)** IMAT formation to collagen content (% of TA area); **C)** Collagen content (% of TA area) to average cross-sectional area normalized to body weight ($\mu m^2/g$). Data for IMAT was obtained from Figure 2, collagen content from Figure 3 and average cross-sectional area normalized to body weight from Figure 4. A Pearson correlation test was used.

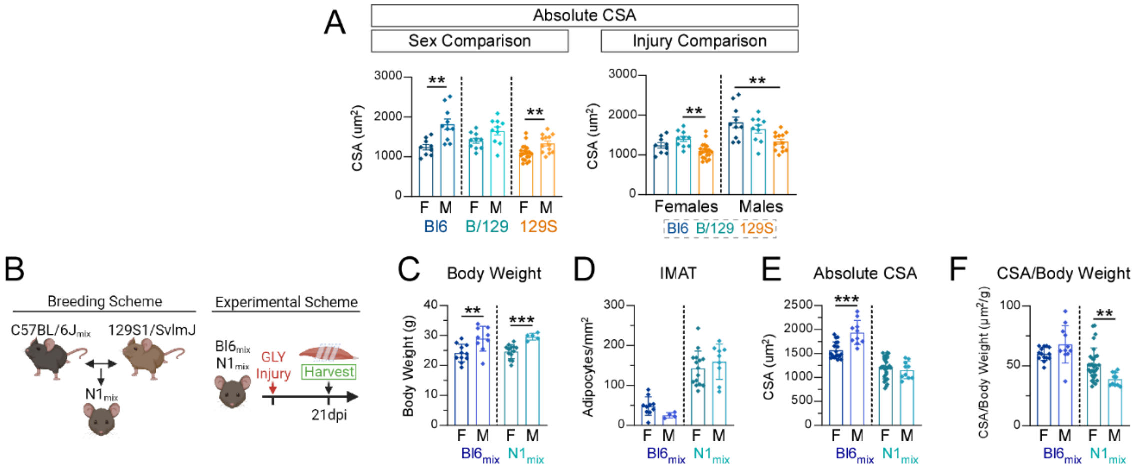

Supplemental Figure 6. Comparison between mixed Bl6 and first generation of 129S cross. A) Average cross-sectional area (CSA; μm^2) of 10-week-old C57BL/6J (Bl6), 129S1/SvlmJ and B6129SF1/J (B/129) males and females. Data are group to compare between: *(Left)* sex within the same strain; *(Right)* strains within the same sex. Data for Bl6 and 129S was obtained from Figure 4. **B)** Breeding and experimental outline. **C)** Body weight (g) of adult, 10-week-old mixed C57BL/6J ($Bl6_{mix}$) and mixed N1 progeny ($N1_{mix}$). **D)** Quantification of adipocytes normalized to injured area ($adipocytes/mm^2$); **E)** average cross-sectional area (CSA; μm^2); and **F)** average CSA normalized to body weight ($\mu m^2/g$) 21 days post GLY injury in $Bl6_{mix}$ and $N1_{mix}$ female and male mice. All data are represented as mean ± SEM. An unpaired two-tailed t test or a one-way ANOVA followed by a Dunnet's multiple comparison was used. A p value less than 0.05 was considered statistically significant where: ** $p \leq 0.01$ and *** $p \leq 0.001$.
